# Fractionated Proton Irradiation Does Not Impair Hippocampal-Dependent Short-Term or Spatial Memory in Female Mice

**DOI:** 10.3390/toxics10090507

**Published:** 2022-08-29

**Authors:** Pilar Simmons, Christa Corley, Antiño R. Allen

**Affiliations:** 1 Division of Radiation Health, University of Arkansas for Medical Sciences, Little Rock, AR 72205, USA; 2 Department of Pharmaceutical Sciences, University of Arkansas for Medical Sciences, Little Rock, AR 72205, USA; 3 Department of Neurobiology and Developmental Sciences, University of Arkansas for Medical Sciences, 4301 W Markham, Little Rock, AR 72205, USA

**Keywords:** space, radiation, hippocampus, behavior

## Abstract

The environment outside the Earth’s protective magnetosphere is a much more threatening and complex space environment. The dominant causes for radiation exposure, solar particle events and galactic cosmic rays, contain high-energy protons. In space, astronauts need healthy and highly functioning cognitive abilities, of which the hippocampus plays a key role. Therefore, understanding the effects of ^1^H exposure on hippocampal-dependent cognition is vital for developing mitigative strategies and protective countermeasures for future missions. To investigate these effects, we subjected 6-month-old female CD1 mice to 0.75 Gy fractionated ^1^H (250 MeV) whole-body irradiation at the NASA Space Radiation Laboratory. The cognitive performance of the mice was tested 3 months after irradiation using Y-maze and Morris water maze tests. Both sham-irradiated and ^1^H-irradiated mice significantly preferred exploration of the novel arm compared to the familiar and start arms, indicating intact spatial and short-term memory. Both groups statistically spent more time in the target quadrant, indicating spatial memory retention. There were no significant differences in neurogenic and gliogenic cell counts after irradiation. In addition, proteomic analysis revealed no significant upregulation or downregulation of proteins related to behavior, neurological disease, or neural morphology. Our data suggests ^1^H exposure does not impair hippocampal-dependent spatial or short-term memory in female mice.

## 1. Introduction

NASA is planning a human round-trip mission to Mars in the 2030s that is anticipated to last roughly 3 years [1]. Long duration excursions beyond low Earth orbit will pose new stressors and risks for the health of the astronaut crew [2]. Once a spacecraft ventures outside of the Earth’s protective magnetosphere the crew will be subjected to an increase in radiation exposure which will require more shielding. Current shielding and attenuation methods are not sufficient for the deep space environment beyond LEO. Before missions outside LEO can take place, there must be guidelines and mitigation strategies established to monitor acceptable risk levels for crewmembers. Expanding our understanding of how radiation exposure will affect the health and performance of crewmembers will provide valuable insight for extended missions beyond LEO.

For human long-term Lunar and Mars explorations, the exposure to episodic solar particle event (SPE) protons and constant fluxes of galactic cosmic rays (GCR) are a major safety concern [3]. High energy protons (^1^H) are the most abundant charged particle in both the SPE and the GCR spectra [4]. Evidence from animal studies suggest highly charged protons from GCR that are able to pass through matter can lead to neuronal injury and degeneration [5]. SPE occur when the sun experiences a solar flare or a coronal mass ejection producing intense periods of increased radiation that can last hours to days [6]. SPE are comprised principally of ^1^H and pose a potentially lethal risk of radiation exposure to astronauts if crew are not sheltered from exposure in a timely manner [5,7].

Hippocampal dentate gyrus (DG) neurogenesis plays an important role in many cognitive functions and is considered a therapeutic target for neurodegenerative diseases [8]. Research has already established a positive correlation between DG neurogenesis and learning and memory in neurologically healthy mice [9,10], and murine data from our laboratory and others show that neurogenic cells are very radiosensitive. Neurogenesis has been shown to decline after administering doses below the threshold for overt tissue injury [11,12]. Furthermore, considerable data now exist showing that decreased neurogenesis is associated with hippocampal-dependent behavioral deficits [13,14]. Data from our laboratory and others have shown that cells in the neurogenic zone of the hippocampal DG are extremely sensitive to low doses of radiation [15,16,17,18] and that such changes are associated with hippocampal-dependent cognitive impairments [19,20]. ^1^H make up a large portion of the deep space radiation environment, making it vital to study how exposures can impact DG neurogenesis. There is data from a study that suggests space radiation exposure does not impact cognitive or behavioral functions of female mice when compared to male [21]. The purpose of this study is to investigate the effects of proton irradiation on hippocampal dependent spatial and short-term memory on female mice.

## 2. Materials and Methods

**Scheme 1:**
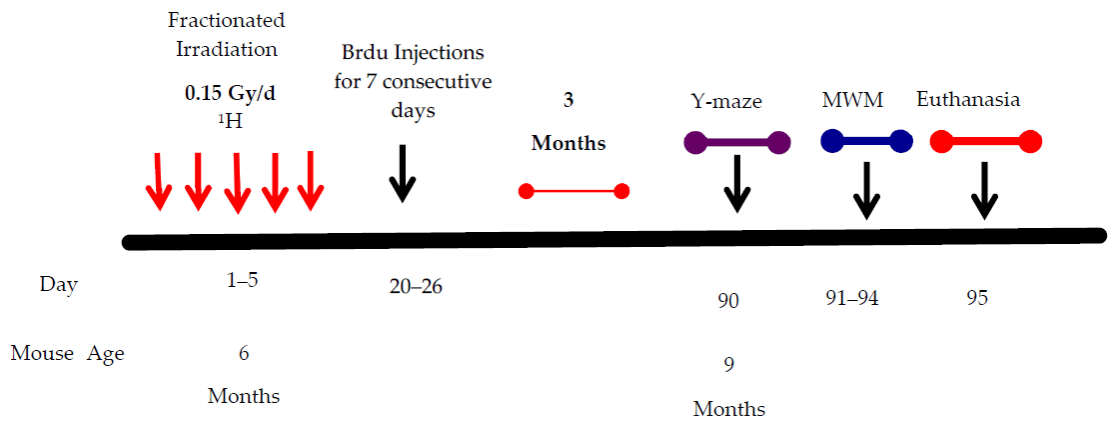
Timeline of experimental design for fractionated irradiation, behaviorial testing and euthanasia.

Eight-week-old female CD1 mice were purchased from Charles rivers (Wilmington, MA, USA) and housed five animals per cage. The mice received standard rodent chow and water ad libitum until they reached 6 months of age. Once the animals were 6 months old, they were transferred to Brookhaven National Laboratory (BNL), where they were acclimated for 1 week before they were exposure. We subjected twenty-four 6-month-old female CD1 mice to 0.75 Gy fractionated ^1^H (250 MeV) whole-body irradiation (0.15 Gy per day for 5 days). Each mouse was placed in a ventilated acrylic box to minimize movement. Holders were then placed in a foam fixture with the long axis of the animal (head to toe) perpendicular to the direction of the beam exposed to whole-body irradiation with a linear energy transfer (LET) of ~0.39 keV/µm. The mice were exposed to a square beam of approximately 20 × 20 cm. Sham-irradiated mice were placed into the plastic enclosures for the same amount of time and on the same day and during the same time of day as the irradiated mice. Dosimetry and beam properties were controlled by NASA Space Radiation Laboratory physicists. After irradiation, animals were transported back to the University of Arkansas for Medical Sciences (UAMS) by overnight airlift and given 2020X chow containing 150 ppm fenbendazole for eight weeks, as a routine UAMS quarantine procedure. Mice remained housed five per cage with Enrich-o’Cob^®^ (The Andersons, Inc., Maumee, OH, USA) bedding. All animal procedures were approved by the Institutional Animal Care and Use Committees of UAMS and Brookhaven National Laboratory. Behavioral and immunohistochemical analyses were recorded in a double-blind analysis by 2 technicians. Treatment groups were assigned, coded, and held until the cellular analyses were complete. Mice were tested for cognitive performance with Y-maze and Morris water maze (MWM) tests 3 months after irradiation (Scheme 1). Only female mice were studied because previous studies were prominently performed on male mice.

### 2.1. Y-Maze

First, mice completed a Y-maze task to assess short-term spatial memory and exploratory activity. The Y-maze is based on the instinctive curiosity of rodents to explore novel stimuli without positive or negative reinforcement. Mice were handled 5 days prior to the onset of behavioral testing. Prior to each day of testing, mice were acclimated to the testing room in their home cage for 1 h. All testing took place during the dark half of the animal’s light/dark cycle. They were exposed in 2 trials to an apparatus composed of 3 arms: start, familiar, and novel. Each clear, acrylic arm (45 × 15 × 30 cm) of the Y-maze contained a unique visual cue affixed to the end of the arm. In the first trial (i.e., training trial), mice were introduced in the start arm (facing the end of the arm) and allowed to explore the start and familiar arms for 5 min; the novel arm was blocked during the training trial. After a 4 h intertrial interval, the mice were reintroduced to the Y-maze for the second trial (i.e., testing trial) and allowed to explore all 3 arms for 5 min.

### 2.2. Morris Water Maze

Hippocampal-dependent spatial learning and memory were assessed with the MWM. The maze is a 5-day task during which the mice are required to locate a hidden platform on the basis of extramaze cues. A circular pool (diameter: 140 cm) was filled with temperature-controlled (24 °C) opaque water. Large, clearly visible cues were located on the test room curtains. The hidden platform did not change quadrants during the task. Each day on days 1 through 5, the mice were tested in 3 sessions with a 2 h intertrial interval, and each session consisted of 3 trials with a 10 min intertrial interval. During acquisition, each mouse was gently placed in the water maze facing the wall from 1 of 7 locations, excluding the location immediately next to the platform (i.e., northeast starting location was excluded for the first test time, and southwest starting location was excluded for the second test time). If the mouse failed to find the platform within the maximum allotted time of 60 s, it was gently placed onto the platform for 20 s.

To measure spatial memory retention, probe trials were conducted following the third session on day 5. For the probe trial, the hidden platform was removed from the pool. Mice were placed in the quadrant opposite the target quadrant, which is the previous location of the hidden platform, and allowed to swim for 60 s. The time spent in the target quadrant was compared to the time spent in the 3 nontarget quadrants. A charged-coupled device video camera was located above the maze for automatic behavioral analysis using EthoVision XT video tracking system (Noldus Information Technology, Wageningen, The Netherlands). Mice were sacrificed on day 5 after the probe trials.

### 2.3. Bromodeoxyuridine Injections

Fourteen days following sham- or ^1^H-irradiation, all mice received daily injections of bromodeoxyuridine (BrdU; 100 mg/kg in saline) for 7 consecutive days. 3 months after the last BrdU injection, mice underwent Y-maze and MWM testing and then were euthanized by cervical dislocation; tissues were collected for analysis of neurogenesis.

### 2.4. Immunofluorescence Staining

#### 2.4.1. Neurogenesis

The brains from multiple animals were blocked together and cryosectioned such that each slide contained sections of 1 animal from each of the experimental groups. Slides were fixed in 2% paraformaldehyde, placed on ice for 8 min, and washed 3 times with Tris-buffered saline (TBS) for 5 min each. Slides were incubated in 2 mol/I hydrochloric acid for 45 min at 37 °C and then neutralized in borate buffer for 10 min. They were washed 8 times with TBS for 5 min each. Tissues were blocked with tyramide signal amplification (TSA) blocking buffer in TBS for 1 h at room temperature then stained with primary antibody rat anti-BrdU (1:400; ab6326, Abcam, Santa Clara, CA, USA) and incubated overnight at room temperature. Slides were then washed in TBS-Tween once for 10 min followed by 2 washes with TBS for 5 min then anti-rat secondary antibody (1:200 Alexa 555; Invitrogen, Waltham, MA USA) for 2 h at room temperature. After washing 3 times with TBS for 5 min each, slides were incubated in immunoglobulin G (IgG) for 1 h at room temperature followed by incubation with mouse-on-mouse (MOM) diluent (M.O.M. Fluorescein Kit, FMK-2201; Vector Laboratories, Newark, CA, USA) for 5 min at room temperature. Slides were then stained in a primary mouse anti-NeuN (MAB377, Millipore Sigma, Temecula, CA, USA) diluted 1:75 and astrocytes with rabbit anti-glial fibrillary acidic protein (GFAP, GA52461-2, Agilent, DaKo, Santa Clara, CA, USA) antibodies diluted 1:200 in MOM (M.O.M. Fluorescein Kit, FMK-2201; Vector) diluent and incubated overnight at 4 °C. They were washed once in TBS-Tween for 10 min. The primary antibody was detected with Alexa Fluor 488 goat anti-mouse IgG antibodies (Thermo Fisher Scientific, Waltham, MA USA) diluted 1:200 and the astrocyte antibody was detected with Alexa Fluor 633 goat anti-rabbit IgG antibodies (Thermo Fisher Scientific) diluted 1:200 in blocking buffer for 2 h at room temperature. The slides preserved with VectaShield (Vector).

#### 2.4.2. Total Activated and Newly Born Activated Microglia

Slides were fixed for 10 min in 4% paraformaldehyde and washed with TBS, then incubated for 30 min in TSA blocking buffer containing 3% normal rabbit serum. Next samples were incubated overnight at 4 °C with rat anti-mouse CD68 antibody diluted 1:1000. Samples were rinsed 5 times in TBS then 2 h at room temperature with rabbit anti-rat IgG (Vector) diluted 1:200. Staining signals were amplified with an avidin/biotin amplification system followed by Cy3 TSA. To label newly born activated microglia, slides were incubated overnight at 4 °C with rat anti-BrdU diluted 1:50 as primary antibody. BrdU was detected with secondary antibody anti-rat fluorescein.

### 2.5. Microscopic Analysis

Briefly, images were collected with a Zeiss AxioImager Apotome microscope using a 20× objective. The parameters were kept constant across sections. Regions of interest were selected using Zeiss imaging software (Carl Zeiss, Hertfordshire, UK) and the numbers of positive cells were counted from both the upper and lower blades of the dentate gyrus of both hippocampi. Manual counts of were performed by an experimenter blind to the experimental conditions. At least four sections per animal were analyzed, from the medial portion of the dorsal hippocampus (from 3.2 to 4.00 mm posterior to bregma). For each section cells were counted (without knowledge of treatments) under high power (×40) using an Apotome Zeiss microscope (Carl Zeiss, Hertfordshire, UK).

### 2.6. Tandem Mass Tag Proteomics Analysis

Tandem mass tag (TMT) technology is a powerful tool for precise and accurate quantitative proteomics. This method has been used widely to characterize protein expression profiles and investigate and compare functional changes at the protein level. The protocol involves extraction of proteins from hippocampal tissues followed by reduction, alkylation, and digestion. Samples from each experimental group were labeled with 1 of 6 isobaric tags of the TMT reagent. Resulting peptides were pooled at equal concentrations before fractionation and data acquisition. The TMT-labeled samples were analyzed by liquid chromatography–mass spectrometry/mass spectrometry (LC–MS/MS). In the first spectrometer scan, or MS1, same-sequence peptides from the different samples appear as a single unresolved additive precursor ion. Fragmentation of the precursor ion during MS/MS, or MS2, yields sequence-informative b- and y-ions, and further fragmentation by synchronous precursor selection (MS3) provides quantitative information as distinct masses between *m*/*z* 126 and 131, representing the “different” reporter ions. The reporter ion intensity indicates the relative amount of peptide in the mixture that was labeled with the corresponding reagent.

### 2.7. Bioinformatics Analysis

Ingenuity Pathway Analysis (IPA; Qiagen, Valencia, CA, USA) was used to investigate affected signaling pathways involving proteins of interest. The ROAST (rotation gene set testing) method was used to investigate unidirectional and bidirectional regulation of significant proteins.

### 2.8. Statistical Analysis

We expressed data as a mean ± the standard error of the mean (SEM). A one-way ANOVA followed by Holms post hoc test were used to evaluate statistical differences between sham and irradiated groups in the Y-maze. Discrimination rations were compared with unpaired *t*-tests with Welch’s corrections. Visible and hidden water maze learning curves were analyzed using 2-way repeated measures ANOVA. The Holm’s correction was used to control for multiple comparisons. Separate analyses were conducted for the visible and hidden platform learning curves. For analysis of performance in the water maze probe trials, one-way ANOVAs were used along with Holms post hoc test, when appropriate. We employed unpaired *t*-tests with Welch’s corrections to evaluate differences in Brdu, neurogenesis, gliogensis and microglia. Differences were considered to be statistically significant when *p* < 0.05. Statistical analyses were conducted with GraphPad Prism 8.0 software (La Jolla, CA, USA).

## 3. Results

### 3.1. Y-Maze

**Figure 1:**
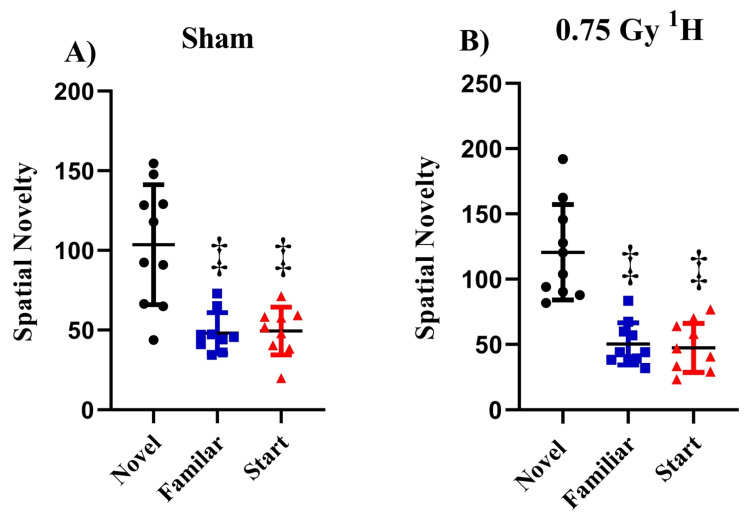
Y-maze. Both sham-irradiated (**A**) and ^1^H-irradiated (**B**) mice significantly preferred exploration of the novel arm compared to the familiar and start arms ** *p* < 0.01.

**Figure 2:**
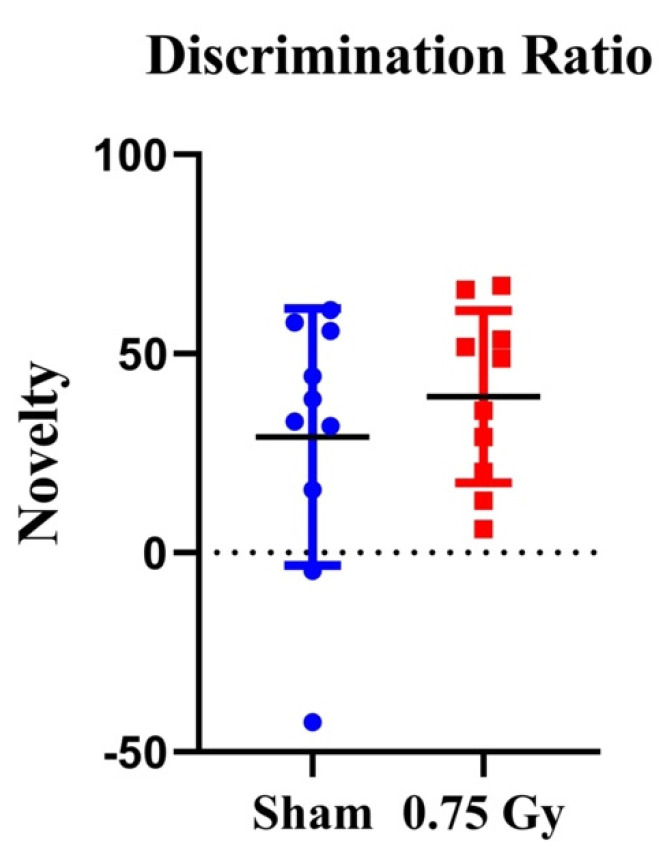
Y-maze discrimination ratio. Sham- and ^1^H-irradiated animals displayed a positive discrimination ratio. There was no difference in discrimination ratios preferred to explore the novel arm.

In the training trial, both sham-irradiated (F_(2, 27)_ = 7.22, *p* < 0.001; Figure 1A) and ^1^H-irradiated (F_(2, 27)_ = 26.37, *p* < 0.0001; Figure 1B) mice significantly preferred exploration of the novel arm compared to the familiar and start arms. Additionally, the discrimination ratio was calculated. Sham- and ^1^H-irradiated mice displayed a positive discrimination ratio. There was no difference in discrimination ratios (t = 0.82, *p* = 0.42); both groups preferred to explore the novel arm (Figure 2), indicating intact short-term spatial memory.

### 3.2. Morris Water Maze

**Figure 3:**
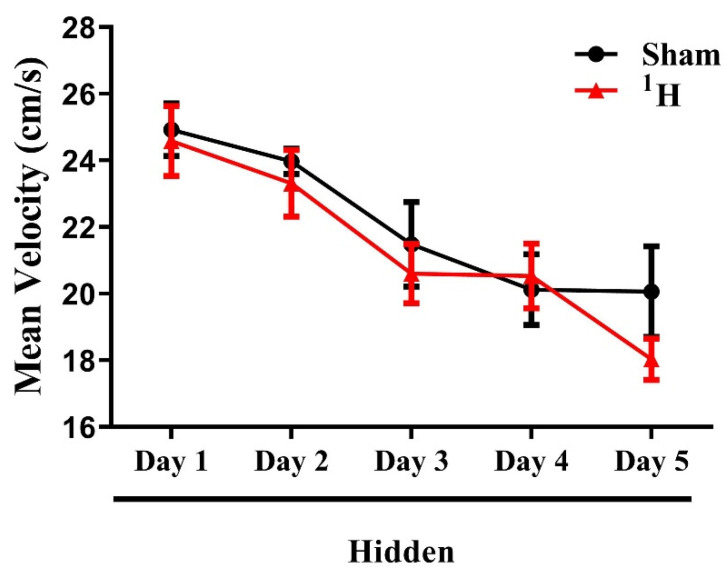
Morris water maze swim velocity. There was no significant treatment-by-day interaction for velocity. Hidden indicates the platform was not visible during the training trials.

**Figure 4:**
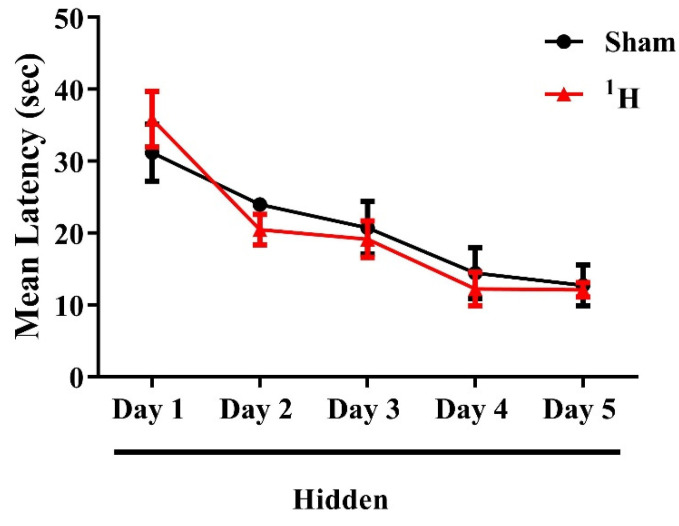
Morris water maze latency. There were no significant differences in treatment-by-day interactions for latency. Hidden indicates the platform was not visible during the training trials.

**Figure 5:**
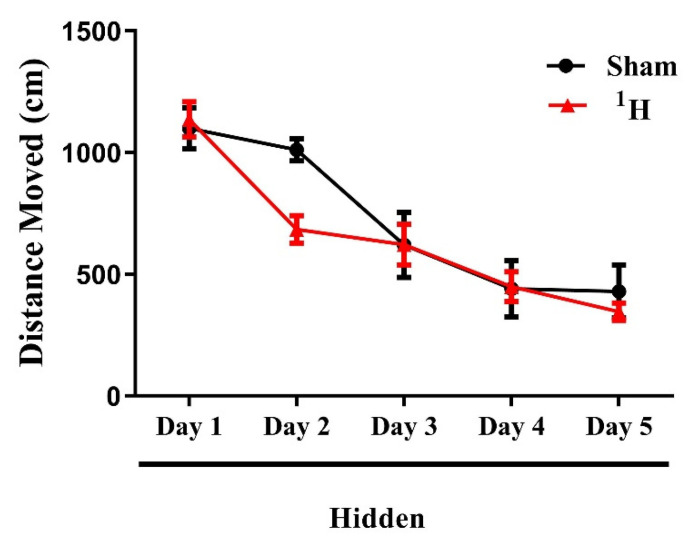
Morris water maze distance moved. There was a significant difference in time (F_(2.9, 52.20)_ = 25.00, *p* < 0.001). Hidden indicates the platform was not visible during the training trials.

Following Y-maze testing, we used the MWM to asses learning and spatial memory retention in the mice. Swim velocity can influence latency to the target during training trials; however, repeated-measures ANOVA revealed that there was no significant treatment-by-day interaction for velocity (F_(4, 72)_ = 0.72, *p* = 0.72, Figure 3). Training trials (i.e., memory acquisition) on days 1 through 5 requires mice to learn the location of the hidden platform based on extramaze spatial cues. The repeated-measures ANOVA revealed no significant differences in treatment-by-day interactions for latency (F_(4, 72)_ = 0.86, *p* = 0.49; Figure 4). However, there was a significant difference in distance moved between treatment groups (F_(2.9, 52.20)_ = 25.00, *p* < 0.001; Figure 5).

**Figure 6:**
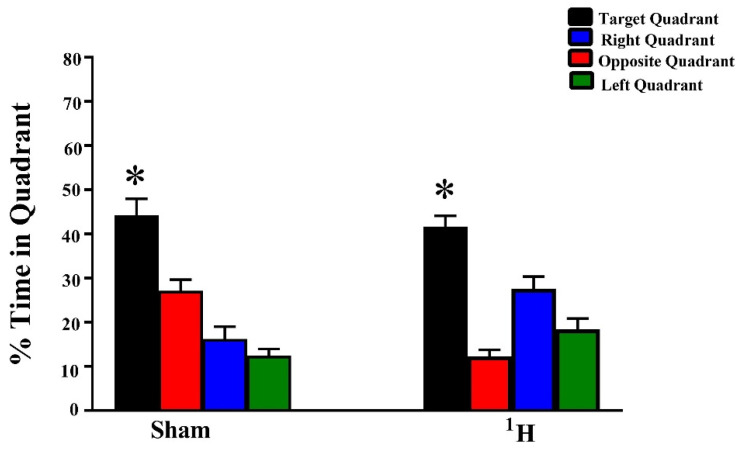
Morris water maze probe trials on day 5. Both the sham- and fractionated ^1^H-irradiated mice spent more time in the target quadrant compared to the right, opposite and left quadrants. * *p* < 0.001.

For probe trial days, normal nonimpaired mice demonstrate spatial memory retention by spending more time in the target quadrant as compared to the other quadrants. The sham-irradiated mice statistically spent more time in the target quadrant compared to the right, opposite, and left quadrants (F_(3, 36)_ = 26.50, *p* < 0.0001). Similarly, ^1^H-irradiated mice also spent more time in the target quadrant compared to the right, opposite, and left quadrants (F_(3, 36)_ = 29.58, *p* < 0.0001; Figure 6).

### 3.3. Neurogenesis and Gliogenesis

**Figure 7:**
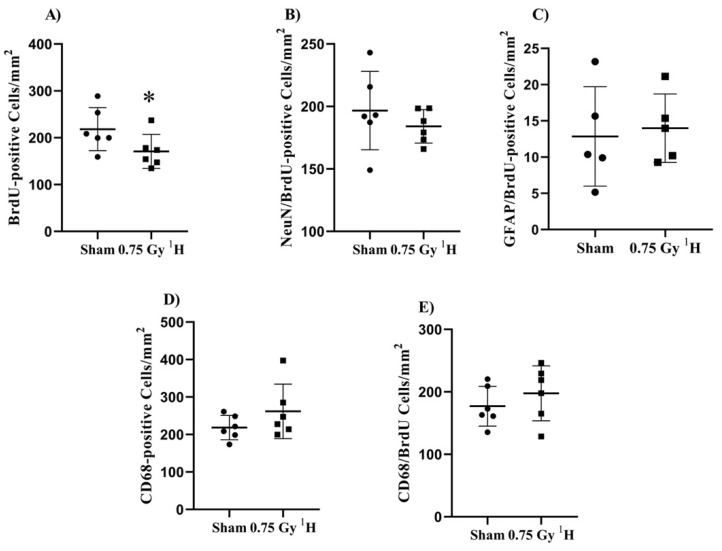
(**A**) Newly born cells. There was a significant difference in the numbers of BrdU^+^ cells in the fractionated ^1^H-irradiated mice compared to sham-irradiated mice (* *p* < 0.05). (**B**) Newly born neurons (NeuN^+^/BrdU^+^). There were no significant differences in the numbers of cells/mm^2^ between groups (*p* = 0.40) ^1^H-irradiated mice (N = 6) compared to sham-irradiated mice (N = 6). (**C**) Newly born astrocytes (GFAP^+/^BrdU^+^). There were no significant differences in the numbers of newly born astrocytes between groups (*p* = 0.69). (**D**) Activated microglia (CD68 only). There were no significant differences in the total numbers of activated microglia between groups (*p* = 0.07). (**E**) Newly born activated microglia (BrdU^+^/CD68^+^). There were no significant differences in the numbers of newly born activated microglia between groups (*p* = 0.15).

The fate of newly born cells was determined by quantifying cells that were double-labeled with BrdU^+^/NeuN for neurons, BrdU^+^/CD68 for activated microglia, and BrdU^+^/GFAP^+^ for astrocytes. The presence of BrdU only represents the long-term survival of newly generated cells, independent of phenotype. ^1^H-irradiated mice had a significantly lower number of BrdU^+^ cells compared to sham-irradiated mice (t = 3.39, *p* < 0.05), with an average of 218.0 + 19.0 cells/mm^2^ in sham-irradiated mice and 170.9 + 14.8 cells/mm^2^ in ^1^H-irradiated mice (Figure 7A). With respect to newly born neurons (BrdU^+^/NeuN^+^), there were no significant differences in the numbers of cells/mm^2^ between groups (t = 0.92, *p* = 0.40), with an average of 196.7 ± 31.2 BrdU^+^/NeuN^+^ cells/mm^2^ in sham-irradiated mice and 184.0 ± 5.5 cells/mm^2^ in ^1^H-irradiated mice (Figure 7B). In addition, there were no significant differences in the numbers of newly born astrocytes (BrdU^+^/GFAP^+^) between groups (t = 0.42, *p* = 0.69), with an average of 12.85 ± 2.1 cells/mm^2^ in sham-irradiated mice and 13.99 ± 2.1 cells/mm^2^ in ^1^H-irradiated mice (Figure 7C).

### 3.4. Total Activated and Newly Born Microglia

There was no significant difference (t = 2.24, *p* = 0.07; Figure 7D) in the total numbers of activated microglia (CD68^+^) in sham-irradiated mice (218.6 ± 13.2 cells/mm^2^) compared to ^1^H-irradiated mice (261.8 ± 29.68 cells/mm^2^). There were no significant differences in the numbers of newly born activated microglia (BrdU^+^/CD68^+^) between groups (t = 1.69, *p* = 0.15), with an average of 177.1 ± 13.0 cells/mm^2^ in sham-irradiated mice and 197.7 ± 17.9 cells/mm^2^ in ^1^H-irradiated mice (Figure 7E).

### 3.5. Proteomics

**Figure 8:**
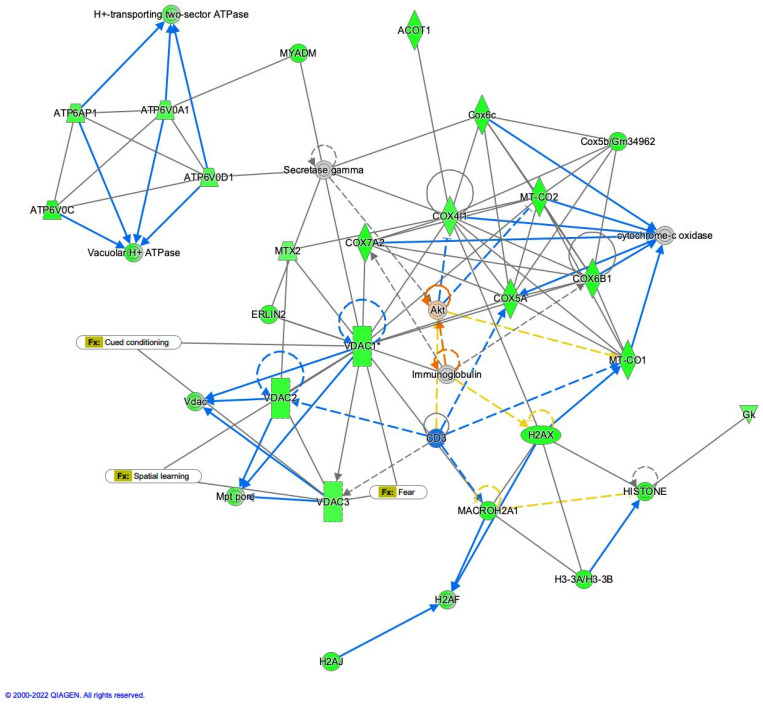
Graphic representation of mouse hippocampus protein network 1, identified by Ingenuity Pathway Analysis (IPA) as being affected by both sham irradiation and ^1^H irradiation. Functions associated with the network include developmental disorder, hereditary disorder, and metabolic disease. The network is overlayed with the disease and function tool to display the 3 key molecules involved with behavior. The node color indicates expression value, and color intensity indicates degree of upregulation (red) or downregulation (green). Gray nodes are dataset molecules that were not significantly expressed and therefore did not pass the IPA analysis cutoff. Uncolored nodes were not part of our dataset but were incorporated into the pathway based on evidence stored in the Ingenuity Knowledge Base. Known direct and indirect interactions between network proteins, as well as the direction of the interaction, are indicated by arrows or blocked lines.

**Figure 9:**
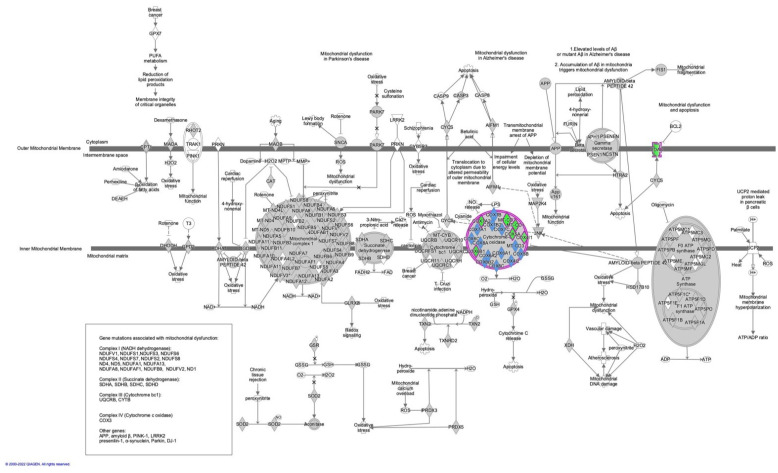
Visual diagram of the mitochondrial dysfunction canonical pathway identified by Ingenuity Pathway Analysis (IPA) as being associated with both the sham-irradiated group and the ^1^H-irradiated group. The diagram provides an image of how the pathway is structured, where the molecules from the dataset are located, and a prediction of molecule activity. The nodes outlined in magenta are the molecules found in the dataset. The node color indicates expression value, and color intensity indicates degree of upregulation (red) or downregulation (green). Gray nodes are dataset molecules that were not significantly expressed and therefore did not pass the IPA analysis cutoff. Uncolored nodes were not part of our dataset but were incorporated into the pathway based on evidence stored in the Ingenuity Knowledge Base. Blue indicates a prediction of inhibited activity. Solid lines indicate a direct interaction between molecules and the predicted relationship between the molecules. Grey arrows indicate the effect is not predicted. The direction of the interaction is represented by arrows. For signaling pathways, an arrow pointing from one molecule to another indicates the cause of activation.

Proteomic analysis was performed to obtain an understanding of the proteins expressed in potential pathways and networks associated with the sham-irradiated group vs. the ^1^H-irradiated group. We conducted IPA with the datasets of differentially expressed proteins and focused on one of the top canonical pathways and the top network. IPA identified mitochondrial dysfunction as the top canonical pathway associated with sham-irradiated mice compared to ^1^H-irradiated mice (Figure 8). The top network had functions associated with developmental disorder, hereditary disorder, and metabolic disease (Figure 9). We used the disease and function overlay tool on the top network to provide insight on potential proteins associated with behavior between the treatment groups (Figures S1 and S2).

## 4. Discussion

In our current study, female mice exposed to ^1^H irradiation showed no difference in discrimination ratios after analysis of Y-maze testing. Both groups preferred to explore the novel arm indicating intact short-term spatial memory. During the MWM task, the mice performed better as testing progressed, suggesting that fractionated ^1^H irradiation does not affect the ability of mice to learn. Both sham- and fractionated ^1^H-irradiated groups also spent more time in the target quadrant during probe trials demonstrating spatial memory retention. Upon examining effects of proton exposure on neurogenesis and gliogenesis, we found no significant differences between sham- and ^1^H-irradiated groups in newly born neurons, in newly born astrocytes, and in activated microglia and newly born activated microglia. Similarly, the IPA results from the proteomic analysis showed no relevant canonical pathways or networks in relation to hippocampal cognition or impairment.

The risk of radiation damage to the central nervous system (CNS) from SPE and GCR exposure is a major concern for human exploration of space. Among the many possible radiation-induced alterations to CNS function, there has been a large focus on the detrimental effects on cognition and behavior. Experimental studies using heavy ion beams simulating space radiation provide constructive evidence of the CNS changes in order to estimate risk [22]. Research with mouse models irradiated with high Z-energy (HZE) nuclei has been demonstrated to induce neurocognitive and behavioral deficits [22]. However, behavioral outcomes suggest differential effects and sensitivity to HZE-particle radiation depending on particle characteristics and energy, strain, sex, age at exposure, and post-irradiation evaluation time [22,23]. As the most abundant particle in SPE and GCR, ^1^H has garnered considerable attention for its effects on the CNS [23]. Similar to HZE particles, results from research on the effects of proton exposure on cognitive performance have also been inconsistent [23]. Several rodent studies examined cognitive performance after proton exposure using behavioral paradigms and showed varying results. A study from Raber et al. investigated hippocampal susceptibility to space radiation-induced changes after proton alone (150 MeV, 0.1 Gy), ^56^Fe alone (600 MeV/n, 0.5 Gy), or combined proton and ^56^Fe irradiation. Male mice exposed to either proton or combined proton irradiation showed impaired novel object recognition 3 months post-irradiation, which was not observed in mice irradiated with ^56^Fe alone [24]. Another study found no significant difference in the amount of time spent with novel object recognition for male rats given whole-body exposures to 150 or 1000 MeV/n protons at doses 0.25, 0.50, or 1 Gy compared to the control rats [25]. A study that exposed adult male mice to 0.5 Gy or 1 Gy ^1^H showed a significant increase in anxiety behavior using open-field testing 9 months post-irradiation [8]. A study conducted by Shukitt-Hale et al. found exposure of male rats to a dose of 1.5 to 4.0 Gy of 250 MeV protons did not significantly disrupt spatial learning and memory as measured by MWM 6 weeks post-irradiation [26].

Alterations in hippocampal neurogenesis are a factor that recent studies suggest might be associated with radiation-induced cognitive impairment [27]. Hippocampal precursors retain the ability to continually proliferate and differentiate to fully mature neurons, including astrocytes, oligodendrocytes, and other glial cells throughout life [28,29,30]. Neurogenesis is reported to have an important role in learning and memory to maintain the cognitive health of an organism [30,31]. Several studies have used rodent models to examine how irradiation impacts hippocampal neurogenesis. Rola et al. irradiated mice with 0.5 to 4 Gy of ^56^Fe ions and 2 months later, quantified neurogenesis and numbers of activated microglia as a measure of neuroinflammation in the DG [29]. Results from this study showed that there were few changes with doses greater than 0.5 Gy, but that there was a dose-related decrease in hippocampal neurogenesis and a dose-related increase in numbers of newly born activated microglia from 0.5 to 4.0 Gy [29].

Another study from this lab irradiated mice with 1 to 3 Gy of ^12^C or ^56^Fe ions and 9 months later, quantified proliferating cells and immature neurons in the dentate subgranular zone in order to observe whether damage to precursor cells would occur [32]. The results showed that reductions in these cells were dependent on the dose and linear energy transfer and that these changes are not only persistent, but also may worsen with time [32]. Another study exposed mice to 1 GeV/n proton radiation at doses of 0 to 2 Gy to determine the time and dose–response characteristics of the CNS to whole-body proton irradiation [33]. These experiments revealed that proton irradiation leads to an acute decrease in cell division within the DG of the hippocampus (with significant differences detected at doses as low as 0.10 Gy), a persistent effect on proliferation in the subgranular zone at 1-month post-irradiation, and a decrease in neurogenesis at doses as low as 0.50 Gy at 3 months post-irradiation [33]. Our data showed no significant decrease in newly born neurons and no significant increase in newly born astrocytes, which suggests no sensitivity to protons at this dose. However, since our dosage was administered in fractions this difference could be due to the priming dose effect. A dose given in increments overtime could prepare the system to respond to the stimulus. Neuroinflammation is characterized by activation of resident microglia and astrocytes and local expression of a wide range of inflammatory mediators [34]. Our data showed no significant increase in activated microglia and no significant increase in newly born activated microglia after proton irradiation. This suggests no inflammatory response; this effect could be associated with the intact special and short-term memory results.

In this study we used proteomic analysis to determine the top canonical pathway and network that may be associated with ^1^H irradiation compared to sham irradiation. We were unable to identify any pathways or networks that significantly expressed proteins related to cognition, behavior, or neural development. The top canonical pathway for our data set was mitochondrial dysfunction. When we overlayed this pathway with the disease and function tool, there were no proteins associated with behavior, neurological disease, or neural cell morphology. Additionally, the top network had no significant proteins associated with hippocampal-related neurological disease or cell morphology. However, 3 behavioral functions, spatial learning, fear, and cued conditioning, appeared to be associated with the protein VDAC (voltage-dependent anion channel). This protein was predicted to be downregulated in both the top network and the top canonical pathway. VDAC is a mitochondrial protein located in the outer mitochondrial membrane and functions as a gatekeeper for the entry and exit of mitochondrial metabolites [35]. VDAC controls crosstalk between mitochondria and the rest of the cell by acting as a key player in mitochondria-mediated apoptosis [35]. VDAC also plays a role in regulating energy metabolism in neurons and studies have shown that VDAC expression is associated with learning and synaptic plasticity in mice [36]. An upregulation of VDAC protein expression could be a response to neuronal injury.

## 5. Conclusions

Our findings indicate no deficits in short term spatial memory after fractionated ^1^H irradiation. However, we investigated the effects of fractionated ^1^H irradiation on female mice and most studies primarily used male mice. There are very few studies comparing the effects of space radiation on male and female mice, especially on the CNS. Among the many factors that can contribute to variations in results, radiation response and sensitivity may differ as a function of sex. Forty percent of the most recent class of astronauts is female [37]. Therefore, it is important to conduct more research comparing both sexes to better understand sex-based differences in space radiation exposure. Our behavioral testing also correlates with our neurogenesis and gliogenesis data. We saw no significant differences in the number of newly born neurons, astrocytes, or activated microglia between sham- and fractionated ^1^H-irradiated mice. In the future it would be ideal to use techniques such as golgi staining to assess how ^1^H irradiation effects dendritic and axonal morphology of neurons and whether it is associated with deficits in cognition. In summary, our work contributes to the few studies on proton exposure and the cognitive impacts on female mice. Previous studies also investigate how an intact hippocampus compared to a hippocampus with lesions is involved in successful recognition memory. One study from Mahnke and colleagues used gene imaging to analyze how parahippocampal areas play a role in memory using human to rat translational tasks [38]. Another study using memory impaired patients found that unlike the medial temporal lobe, the hippocampus is not needed for scene construction, shifts in perspective or perceiving the geometry of scenes [38]. For future studies it would be beneficial to take these findings in consideration and compare the translational link between rodents to humans.

## Data Availability

The mass spectrometry proteomics data have been deposited to the ProteomeXchange Consortium via the PRIDE partner repository. **Project Accession:** PXD035933. **Project DOI:** 10.6019/PXD035933. **Reviewer Account Details: Username:** reviewer_pxd035933@ebi.ac.uk. **Password:** Kc2c0DWy.

## Supplementary Materials

The following supporting information can be downloaded at: https://www.mdpi.com/article/10.3390/toxics10090507/s1, Figure S1: IPA Legend; Figure S2: IPA Network Shapes.
