# Supplementary material for: Fractionated Proton Irradiation Does Not Impair Hippocampal-Dependent Short-Term or Spatial Memory in Female Mice
Source: Toxics. 2022 Aug 29;10(9):507. doi: 10.3390/toxics10090507 (PMC9503909; doi:10.3390/toxics10090507)
Supplement: Supplementary file 1 [file toxics-10-00507-s001.zip › toxics-1744980-supplementary.pdf]

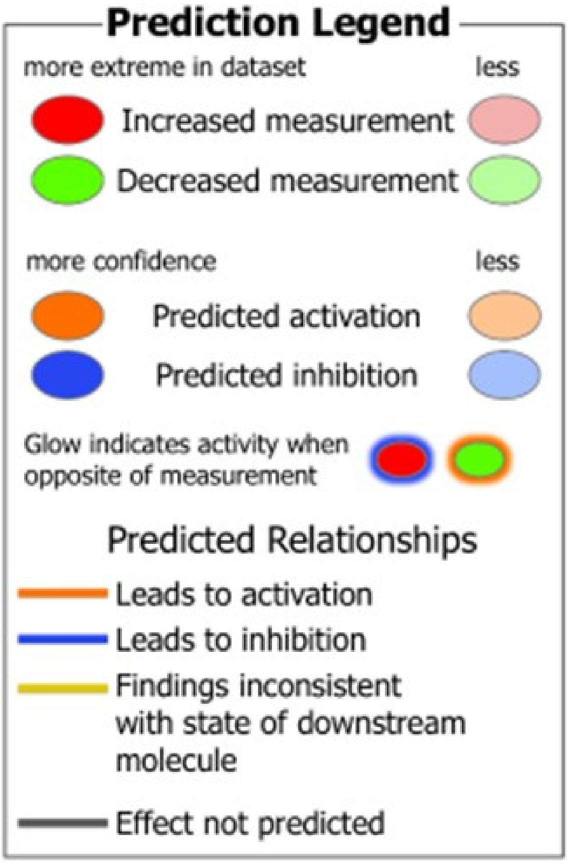

Figure S1. IPA legend

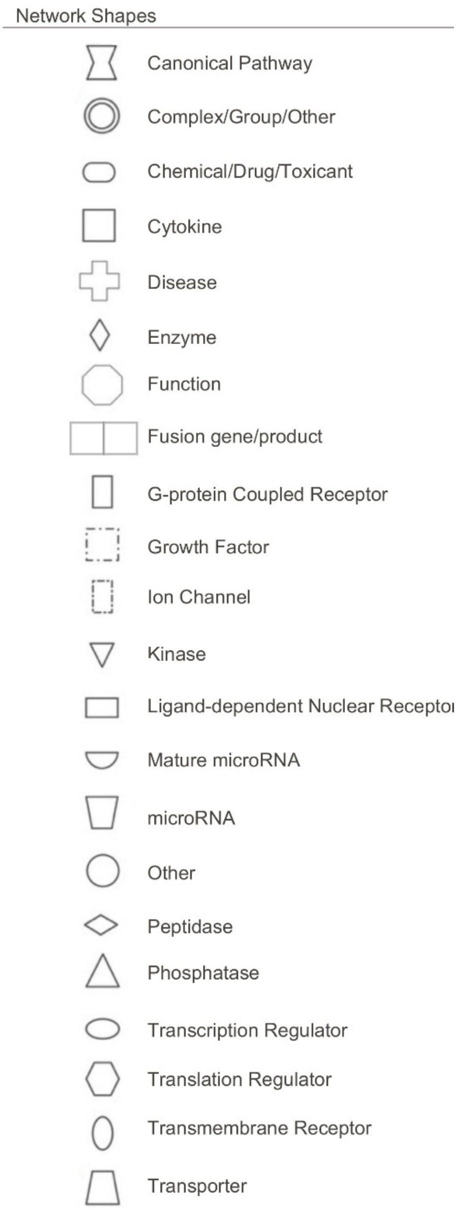

Figure S2. IPA Network Shapes.
